# Geography of Fracture Incidence in Postmenopausal Women with Osteoporosis Treated with Abaloparatide

**DOI:** 10.1007/s00223-017-0375-z

**Published:** 2017-12-28

**Authors:** Michael R. McClung, Gregory C. Williams, Gary Hattersley, Lorraine A. Fitzpatrick, Yamei Wang, Paul D. Miller

**Affiliations:** 1 Oregon Osteoporosis Center, Portland, OR 97210 USA; 2 Institute of Health and Ageing, Australian Catholic University, Melbourne, Australia; 3 Radius Health, Inc., Waltham, MA USA; 4 Panorama Orthopedics and Spine Center, Golden, CO USA

**Keywords:** Abaloparatide, Fracture, FRAX, Geography, Osteoporosis

## Abstract

Geographic heterogeneity has been observed in fracture risk and efficacy of therapeutic intervention in postmenopausal osteoporosis. The objectives of these analyses were to assess across geographic and ethnic subgroups the heterogeneity of fracture incidence and baseline risk, and consistency of effect of abaloparatide-SC vs placebo on fracture risk reduction in the 18-month, phase 3, multinational, ACTIVE randomized controlled trial. Prespecified exploratory analyses of geographic subgroups (North America, South America, Europe, Asia) and post hoc analyses of ethnic subgroups (Hispanic or Latino, other) of postmenopausal women with osteoporosis enrolled in the abaloparatide-SC and placebo cohorts (*n* = 1645) were performed. Country-specific FRAX models were used to calculate 10-year absolute fracture risks. Relative risk reductions for vertebral fractures and hazard ratios for non-vertebral, clinical, and major osteoporotic fractures were calculated. Forest plots were constructed to assess treatment-by-subgroup interactions for each geographic region and ethnicity. Baseline prevalence of vertebral fractures was similar across geographies; baseline prevalence of non-vertebral fractures was more variable. Ten-year major osteoporosis fracture and hip fracture risks were variable across and within regions. The effects of abaloparatide-SC on reducing the risk of vertebral, non-vertebral, clinical, and major osteoporotic fractures were similar across regions, and for Hispanic or Latino vs other ethnicities. A limitation was the limited power to detect interactions with few events. In conclusion, despite geographic variability in fracture incidence and risk at baseline, no differences were detected in the effects of abaloparatide-SC in reducing vertebral, non-vertebral, clinical, and major osteoporotic fracture risk across assessed geographic regions and ethnicities.

## Introduction

Abaloparatide is a 34-amino acid peptide that selectively binds to the parathyroid hormone receptor type 1 (PTHR1) with high affinity and selectivity for the G protein-dependent RG receptor conformation and demonstrates potent anabolic activity with less bone resorption and improved temperature stability compared with teriparatide [1]. In the multinational phase 3 Abaloparatide Comparator Trial in Vertebral Endpoints (ACTIVE) trial, postmenopausal women with osteoporosis were randomized to receive double-blind daily subcutaneous injections of abaloparatide (abaloparatide-SC) or placebo or open-label teriparatide for 18 months at 28 sites in 10 countries. Abaloparatide-SC increased bone mineral density (BMD) and decreased the risk of vertebral and non-vertebral fractures compared with placebo and was well-tolerated [2]. Abaloparatide-SC also increased BMD earlier at non-vertebral sites and decreased the risk of major osteoporotic fractures (hip, forearm, proximal humerus, or clinical spine) compared with teriparatide.

Regional and national registration studies have demonstrated a greater than 10-fold difference in hip fracture rates in 63 countries around the world [3]. Additionally, in 40 countries with FRAX models to estimate the 10-year absolute risk of fracture, there was a similar heterogeneity in the risk of major osteoporotic fractures [3]. A recent report suggested that geographic heterogeneity in the risk of non-vertebral fractures may have contributed to the lack of non-vertebral risk reduction in a large phase 3 trial with romosozumab, an anabolic agent with a different mechanism of action than abaloparatide-SC [4]. Fracture incidence and the effectiveness of romosozumab therapy were lower in patients from Latin America than in those from the rest of the world. Additionally, several analyses have demonstrated variability in the interaction of treatment effects when pre-treatment fracture probabilities were assessed by FRAX [5]. Given the multinational character of the ACTIVE trial, we have investigated the fracture incidence and risk at baseline in these different geographic regions, and the consistency of effect of abaloparatide-SC on fracture reduction.

## Methods

### Subjects

The multicenter, multinational ACTIVE study enrolled postmenopausal women, ages 49–86 years, with osteoporosis as defined by prior radiographic vertebral fracture or recent (within 5 years of enrollment) non-vertebral fracture with a BMD T-score ≤− 2.5 at the lumbar spine or femoral neck if age ≤ 65 years or ≤ − 2.0 if age > 65 years. For those aged > 65 years, no prior fracture was required if the lumbar spine or femoral neck BMD *t* score was ≤− 3.0. Other inclusion/exclusion criteria have been previously described [2]. The protocol was approved by the respective institutional review boards.

### Procedures

After informed written consent was obtained; subjects were screened and then randomized 1:1:1 to receive either blinded daily injections of abaloparatide-SC 80 µg, matching placebo, or open-label daily subcutaneous injections of teriparatide 20 µg for 18 months. These analyses include the 1645 women in ACTIVE who received abaloparatide-SC or placebo. All subjects received supplements of 500–1000 mg/day elemental calcium and 400–800 IU vitamin D based on regional standard of care. The endpoints were assessed as previously described [2], including the primary endpoint of the incidence of new radiographic vertebral fractures from baseline to 18 months in subjects treated with abaloparatide-SC compared to placebo. Non-vertebral fractures, a secondary endpoint, were initially self-reported and then verified from source documents and excluded those of the spine, sternum, patella, toes, fingers, skull, and face and those associated with high trauma, defined as a fall from a height equal to or higher than the level of a stool, chair, or first rung of a ladder or those associated with severe trauma other than a fall. Prespecified exploratory endpoints included clinical fractures (all fractures that would cause a patient to seek medical care, regardless of the level of trauma, including clinical spine fractures) and major osteoporotic fractures (upper arm, forearm, hip, or clinical spine fractures). Study oversight was performed and safety was assessed as previously described [2], and all assessors of fracture were blinded to treatment assignment.

### Statistical Analyses

To evaluate whether the effects of abaloparatide-SC vs placebo on vertebral, non-vertebral, clinical, and major osteoporotic fractures were consistent in different geographical regions, these endpoints were analyzed by subgroups prespecified by the regions of North America, South America, Europe, and Asia, as previously described for other subgroups [6]. Relative risk ratios for new vertebral fractures and hazard ratios for all other fractures were calculated as previously described [2]. Forest plots were constructed to assess qualitative treatment-by-subgroup interactions, and this was further assessed qualitatively and quantitatively by the Breslow–Day test for vertebral fractures and the Cox proportional hazards model for non-vertebral, clinical, and major osteoporotic fractures. Analyses for interactions were not controlled for covariates other than subgroup, treatment, and treatment-by-subgroup interaction in the Cox model. The consistency of the effect of abaloparatide-SC vs placebo on vertebral, non-vertebral, clinical, and major osteoporotic fractures was tested in post hoc analyses in Hispanic or Latino and in non-Hispanic or Latino populations from all geographies. *P* values were not adjusted for multiple comparisons and were considered significant if *p* < 0.05.

To evaluate absolute fracture risk at baseline, the prespecified exploratory FRAX analyses were performed as previously described [5]. FRAX models were used for each country including the United States in North America; Argentina and Brazil in South America; the Czech Republic, Denmark, Estonia, Lithuania, Poland, and Romania in Europe; and Hong Kong in Asia. Ethnic-specific models were used in the United States. The baseline clinical risk factors of age, BMI, prior fragility fracture, current tobacco smoking, ever long-term use of oral glucocorticoids, rheumatoid arthritis, other causes of secondary osteoporosis, and daily consumption of 3 or more units of alcohol were entered into the country-specific FRAX models to calculate the 10-year probability of major osteoporotic fracture and hip fracture with and without the inclusion of femoral neck BMD using the FRAX tool (http://www.shef.ac.uk/FRAX/). A prior fracture was considered present in the FRAX model if there was either a history of prior non-vertebral fracture or a vertebral fracture of semiquantitative grade 2 or 3 documented by radiography at baseline. Current tobacco smoking was assumed if the subjects responded positively to the question of smoking in the last 5 years. Ever long-term use of oral glucocorticoids was set to no for all subjects, as this was an exclusion criterion for the study. As parental history of hip fracture was not captured, this risk factor was set to no. A history of rheumatoid arthritis was captured from the medical history. The diagnosis of secondary osteoporosis was based on the medical history and comprised type 1 diabetes mellitus, malnutrition, liver disorders, and premature menopause. Daily consumption of alcohol was reported if the subject consumed 21 or more units per week.

## Results

The baseline characteristics for each region are presented in Table 1. The women in South America and Asia were slightly older than those in North America and Europe. As expected, the BMI was lower in women from Asia than those from other regions. Among 398 Hispanic or Latino women in the study population, 338 (85%) were white. The baseline prevalence of vertebral fractures was similar across geographies, ranging from 18.0% in South America to 25.3% in Europe. However, the baseline prior history of non-vertebral fractures was more variable, ranging from 32.2% in Asia to 80.0% in North America. At baseline in the overall population, the 10-year risk of major osteoporotic fracture (± SD) was 13.2 ± 7.9% and that for hip fracture was 4.8 ± 4.7% in the FRAX model including BMD and 13.3 ± 8.1% and 5.1 ± 4.8%, respectively, in the model without BMD. Using the FRAX model with BMD, the 10-year major fracture risks ranged from 8.8% in South America to 17.7% in Asia and risks for hip fracture ranged from 3.7% in South America to 8.3% in Asia, but the variability was high (Table 1). There was also variability within regions. For example, within Europe, the 10-year hip fracture risk ranged from 1.6% in Romania to 7.0% in Denmark and in South America the risk was 3.6% in Brazil and 5.2% in Argentina. The subgroups were well matched across the placebo and abaloparatide-SC groups, although some of the subgroups were small (Table 1).

**Table 1:** Patient demographics and disease characteristics, by Region and Treatment Group

	North America^a^	South America			Europe			Asia		
	Total *N* = 30	PBO *N* = 217	ABL-SC *N* = 222	Total *N* = 439	PBO *N* = 461	ABL-SC *N* = 460	Total *N* = 921	PBO *N* = 130	ABL-SC *N* = 125	Total *N* = 255
Age (years) [mean (SD)]	63.8 (6.9)	71.1 (5.7)	71.4 (6.0)	71.2 (5.8)	66.8 (6.3)	67.3 (6.5)	67.0 (6.4)	71.6 (5.5)	71.2 (5.3)	71.4 (5.4)
BMI (kg/m^2^) [mean (SD)]	25.8 (3.2)	25.5 (3.7)	25.5 (3.6)	25.5 (3.6)	25.6 (3.4)	25.4 (3.4)	25.5 (3.4)	22.3 (2.4)	22.7 (2.9)	22.5 (2.7)
Race *n* (%)										
White	27 (90.0)	183 (84.3)	188 (84.7)	371 (84.5)	460 (99.8)	460 (100)	920 (99.9)	0	0	0
Asian	0	1 (0.5)	3 (1.4)	4 (0.9)	0	0	0	130 (100)	125 (100)	255 (100)
Black or African American	1 (3.3)	23 (10.6)	25 (11.3)	48 (10.9)	0	0	0	0	0	0
Other	2 (6.7)	10 (4.6)	6 (2.7)	16 (3.6)	1 (0.2)	0	1 (0.1)	0	0	0
Hispanic or Latino ethnicity [n (%)]	24 (80.0)	181 (83.4)	186 (83.8)	367 (83.6)	5 (1.1)	2 (0.4)	7 (0.8)	0	0	0
BMD T-score [mean (SD)]										
Total hip	− 1.9 (0.6)	− 2.0 (0.8)	− 2.0 (0.7)	− 2.0 (0.8)	− 1.7 (0.7)	− 1.7 (0.7)^b^	− 1.7 (0.7)^c^	− 2.4 (0.6)^d^	− 2.3 (0.7)	− 2.3 (0.6)^e^
Femoral neck	− 2.4 (0.6)	− 2.2 (0.7)	− 2.3 (0.6)	− 2.2 (0.6)	− 2.0 (0.6)	− 2.0 (0.6)^b^	− 2.0 (0.6)^c^	− 2.8 (0.5)^d^	− 2.6 (0.6)	− 2.7 (0.5)^e^
Lumbar spine	− 2.7 (0.9)	− 3.2 (0.8)	− 3.1 (0.9)	− 3.1 (0.9)	− 2.8 (0.8)	− 2.7 (0.9)^f^	− 2.7 (0.8)^g^	− 3.1 (0.8)	− 3.1 (0.7)	− 3.1 (0.8)
Prevalent vertebral fracture [n (%)]	6 (20.0)	39 (18.0)	40 (18.0)	79 (18.0)	125 (27.1)	108 (23.5)	233 (25.3)	23 (17.8)^d^	24 (19.2)	47 (18.5)^e^
Prior non-vertebral fracture [n (%)]	24 (80.0)	86 (39.6)	90 (40.5)	176 (40.1)	280 (60.7)	259 (56.3)	539 (58.5)	39 (30.0)	43 (34.4)	82 (32.2)
10-year absolute fracture risk (%) [mean (SD)]^h^										
Major osteoporotic fracture	16.5 (6.4)	8.6 (4.5)	9.0 (5.0)	8.8 (4.7)	13.9 (7.9)	13.9 (8.1)	13.9 (8.0)	17.8 (8.0)	17.5 (9.6)	17.7 (8.8)
Hip fracture	4.0 (3.2)	3.5 (2.9)	3.9 (3.6)	3.7 (3.3)	4.4 (4.2)	4.5 (4.3)	4.4 (4.2)	8.3 (5.6)	8.2 (7.6)	8.3 (6.6)

^a^North American treatment groups not shown because of small number of subjects in whom no incident fractures occurred

^b^*n* = 458; ^c^*n* = 919; ^d^*n* = 129; ^e^*n* = 254; ^f^*n* = 459; ^g^*n* = 920; ^h^Using the FRAX model with BMD. Two patients in Europe and 1 in Asia did not have baseline femoral neck BMD determinations, and their FRAX scores were not included

**Fig. 1:**
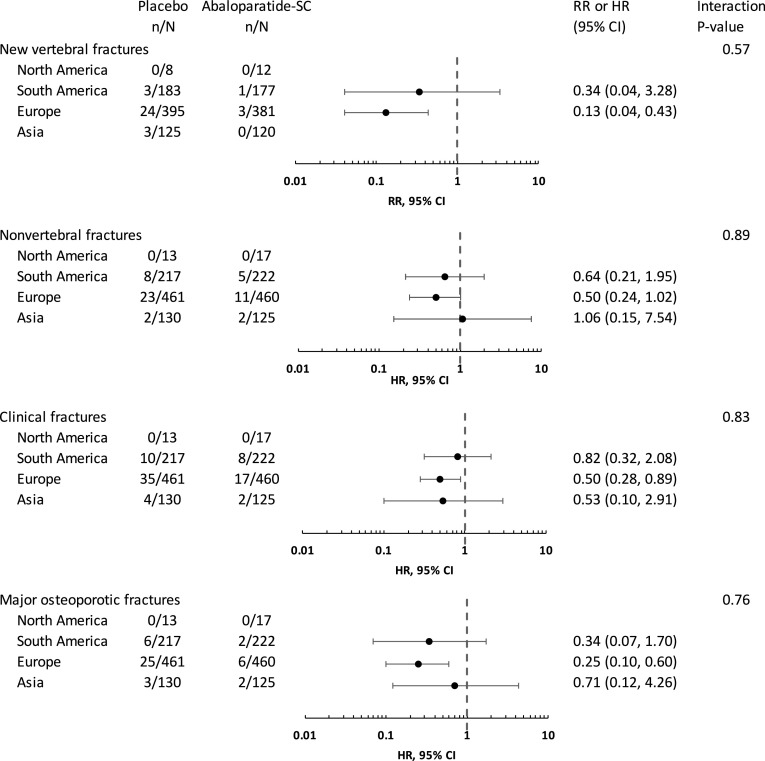
Relative risk ratios for new vertebral fractures and hazard ratios for non-vertebral, clinical, and major osteoporotic fractures in placebo vs abaloparatide-SC groups by geographical region. *RR* relative risk, *HR* hazard ratio; *CI* confidence interval

In the overall population, abaloparatide-SC reduced the risk of vertebral fractures from 4.2% in the placebo group to 0.6% in the treatment group [relative risk (RR) 0.14, 95% confidence interval (CI) 0.05–0.39, *p* < 0.001] [2]. As shown in the Forest plot (Fig. 1), the effect of abaloparatide-SC in reducing the risk of vertebral fractures was similar in South America, Europe, and Asia, although the numbers of events were small in South America and Asia and there were no new vertebral fractures in subjects from North America. There was no significant treatment-by-subgroup interaction (*p* = 0.57).

Overall, abaloparatide-SC reduced the risk of non-vertebral fractures from 4.7% in the placebo group to 2.7% in the treatment group [hazard ratio (HR) 0.57, 95% CI 0.32–1.0, *p* = 0.049] [2]. The Forest plot (Fig. 1) shows similar effects in South America and Europe; there were few events in Asia and none in North America. Again, there was no significant treatment-by-subgroup interaction (*p* = 0.89).

Similar results were found for the prespecified exploratory endpoints of clinical fractures and major osteoporotic fractures. Overall, abaloparatide-SC reduced the risk of clinical fractures from 8.3% in the placebo group to 4.0% in the treatment group (HR 0.57, 95% CI 0.35–0.91, *p* = 0.02) and reduced the risk of major osteoporotic fractures from 6.2% in the placebo group to 1.5% in the treatment group (HR 0.30, 95% CI 0.15–0.61, *p* < 0.001) (2). The Forest plot (Fig. 1) again demonstrates similar effects across regions with no fractures in North America and no significant treatment-by-subgroup interactions (*p* = 0.83 and *p* = 0.76 for clinical fractures and major osteoporotic fractures, respectively).

**Fig. 2:**
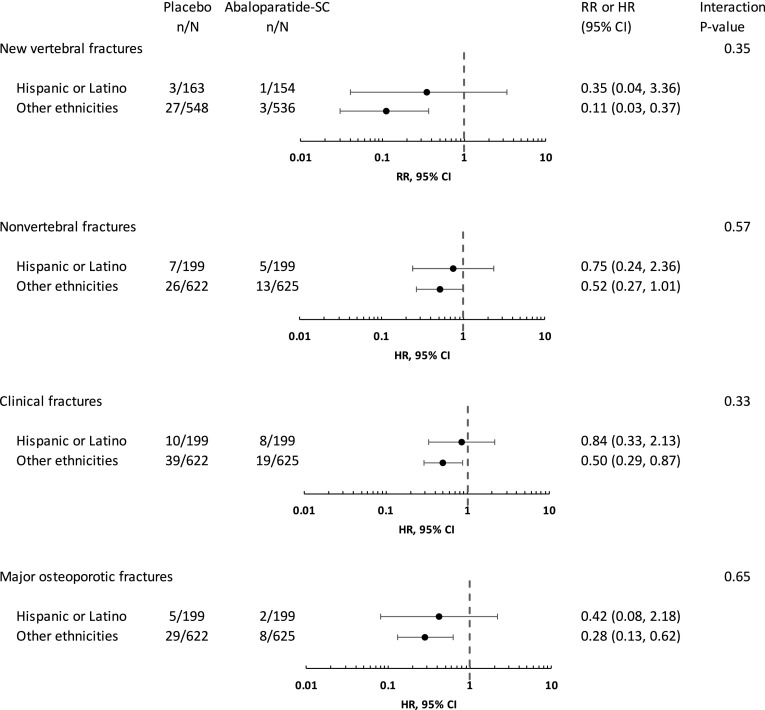
Relative risk ratios for new vertebral fractures and hazard ratios for non-vertebral, clinical, and major osteoporotic fractures in placebo vs abaloparatide-SC groups by ethnicity. *RR* relative risk, *HR* hazard ratio, *CI* confidence interval

The effect of abaloparatide-SC in reducing the risk of vertebral fractures was similar in the Hispanic or Latino population (RR 0.35, 95% CI 0.04–3.36) vs other ethnicities (RR 0.11, 95% CI 0.03–0.37), interaction *p* = 0.35 (Fig. 2). Likewise, the effect of abaloparatide-SC on reducing the risk of non-vertebral fractures was similar in the Hispanic or Latino population (HR 0.75, 95% CI 0.24–2.36) vs other ethnicities (HR 0.52, 95% CI 0.27–1.01), interaction *p* = 0.57. There were also no significant treatment-by-subgroup interactions for the prespecified exploratory endpoints of clinical fractures and major osteoporotic fractures (*p* = 0.33 and 0.65, respectively).

## Discussion

No differences were detected in the effects of abaloparatide-SC in reducing the risks of fracture across the four geographical regions assessed. These comparable effects were achieved for vertebral, non-vertebral, clinical, and major osteoporotic fractures despite the variability in fracture incidence and in the FRAX-derived estimates of fracture risk observed across regions and even across countries within specific regions. Although the same inclusion criteria were used for enrollment in all geographic regions, fracture incidence was higher in European compared to South American patients, consistent with the higher FRAX estimates of fracture probability in the Europe subgroup than in the South American subgroup. This variability in fracture risk has been documented in the past and may be related more to environmental rather than to genetic factors [3]. These results are consistent with a post hoc analysis demonstrating that the efficacy of abaloparatide-SC to decrease the risk of major osteoporotic fracture or any clinical fracture in postmenopausal women with low BMD and/or prior fracture appears independent of baseline fracture probability [5].

Similar results have been found with denosumab for effects on vertebral fracture across the regions of Western Europe/Australia/New Zealand, Eastern Europe, Latin America, and North America [7]. Likewise, the effects of zoledronic acid on vertebral, hip, and non-vertebral fractures were independent of the regions of the Americas, Asia, and Europe [8]. However, in a post hoc analysis, the effect of romosozumab on non-vertebral fracture risk was different in Latin America compared to other regions [4]. Analyses of geographic variations in the results of randomized, controlled trials suggest that although most geographic variations are likely due to chance, some may represent true benefit or harm of the intervention and that the overall average result of a trial is usually a more reliable estimate of the treatment effect in the subgroups than the observed effects in individual subgroups [9]. Consistency of results with those seen in other trials of the same or similar interventions has been suggested as one criterion to guide interpretation of a trial that shows variations in treatment effects across geographies [9].

The majority of subjects in both North America and South America were of Hispanic or Latino ethnicity. Abaloparatide-SC also reduced the risk of fracture to a similar degree in women of Hispanic or Latino ethnicity compared to those of other ethnicities. In the United States, rates of fracture are lower in Hispanic women than in white women, although there is some variability by fracture site [10]. Additionally, the Hispanic community is diverse, and there are regional differences in hip fracture incidences for Hispanics in the United States [11].

The strengths of this study include the diverse populations studied and the prespecified analyses of regional subgroups. The major limitation is that the study was not powered to detect interactions in small groups with few events and wide confidence intervals. Therefore, these analyses cannot exclude the possibility of a treatment effect in some subgroups, especially in the small Asian subgroups. Another important limitation is possible selection bias in that clinical trial subjects may not be truly representative of the general population. Additionally, statistical significance was assessed without adjustment for multiple comparisons.

In conclusion, in postmenopausal women with osteoporosis, no differences were detected in the effects of abaloparatide-SC in reducing vertebral, non-vertebral, clinical, and major osteoporotic fracture risk across different geographic regions and in different ethnicities, despite the geographical variability in baseline fracture incidence and risk.
