# Correction to: Geography of Fracture Incidence in Postmenopausal Women with Osteoporosis Treated with Abaloparatide

**DOI:** 10.1007/s00223-018-0390-8

**Published:** 2018-01-31

**Authors:** Michael R. McClung, Gregory C. Williams, Gary Hattersley, Lorraine A. Fitzpatrick, Yamei Wang, Paul D. Miller

**Affiliations:** 1 Oregon Osteoporosis Center, Portland, OR 97210 USA; 2 Institute of Health and Ageing, Australian Catholic University, Melbourne, Australia; 3 Radius Health, Inc., Waltham, MA USA; 4 Panorama Orthopedics and Spine Center, Golden, CO USA

## Correction to: Calcified Tissue International 10.1007/s00223-017-0375-z

The article Geography of Fracture Incidence in Postmenopausal Women with Osteoporosis Treated with Abaloparatide, written by Michael R. McClung, Gregory C. Williams, Gary Hattersley, Lorraine A. Fitzpatrick, Yamei Wang, Paul D. Miller, was originally published electronically on the publisher’s internet portal (currently SpringerLink) on 28 December 2017 without open access.

With the author(s)’ decision to opt for Open Choice the copyright of the article changed on 8 January 2018 to © The Author(s) 2018 and the article is forthwith distributed under the terms of the Creative Commons Attribution 4.0 International License (http://creativecommons.org/licenses/by/4.0/), which permits use, duplication, adaptation, distribution and reproduction in any medium or format, as long as you give appropriate credit to the original author(s) and the source, provide a link to the Creative Commons license and indicate if changes were made.

The original article has been corrected.

